# Formación docente y percepción del TDAH en el ámbito escolar

**DOI:** 10.1016/j.aprim.2026.103451

**Published:** 2026-02-03

**Authors:** Edwin Gustavo Estrada-Araoz

**Affiliations:** Universidad Nacional Amazónica de Madre de Dios, Puerto Maldonado, Perú

*Sr. Editor,*

Leí con atención el artículo «Análisis del déficit de atención en el TDAH en la población escolar»^1^, un tema de interés también para los profesionales de atención primaria debido al papel que desempeñan en la detección y la derivación de posibles casos. El estudio analiza la coincidencia entre padres y profesores en la identificación de síntomas de inatención y muestra datos concretos sobre cómo se evalúan estos comportamientos en el hogar y en la escuela. La muestra amplia y el uso de una escala reconocida dan al trabajo un respaldo consistente para comprender cómo se perciben estas dificultades en distintos entornos. Además, la diferencia en las prevalencias reportadas por ambos grupos de informantes abre un campo de discusión importante en torno a la detección temprana del TDAH en el contexto escolar.

Al revisar el trabajo, surge un punto que merece atención: el rol que cumple la formación docente en salud mental infantil. La forma en que un profesor interpreta la conducta de un estudiante con posible TDAH no depende solo de lo que ocurre en el aula, sino también del nivel de preparación que tenga sobre el trastorno. Se ha observado que la formación específica, especialmente la vinculada a necesidades educativas especiales, fortalece el dominio docente de los síntomas, el diagnóstico y las opciones de intervención en TDAH, lo que influye directamente en la manera en que estas conductas son reconocidas e interpretadas^2^. En muchos programas de formación inicial docente estos contenidos aparecen de manera superficial o no se actualizan con la frecuencia necesaria^3^. Esto puede generar diferencias en la forma en que los docentes valoran la inatención, más aún en grupos numerosos o en aulas con altas demandas académicas.

Si la formación docente es diversa y, en algunos casos, insuficiente, las cifras más altas reportadas por los docentes podrían no reflejar necesariamente una mayor prevalencia real, sino una interpretación influida por su nivel de formación y por las condiciones del aula. Además, factores como la carga laboral, la cantidad de estudiantes por aula o la presión curricular podrían intensificar la percepción de inatención en determinados contextos. Por ello, profundizar en este aspecto podría orientar futuros estudios e incluso esclarecer parte de la diferencia observada entre padres y docentes.

Este punto también abre la posibilidad de pensar en acciones concretas. Incluir contenidos sobre TDAH y otros trastornos del neurodesarrollo en la formación inicial, ofrecer espacios de actualización periódica, crear equipos de trabajo entre psicólogos escolares y docentes, o facilitar asesorías para casos que generan dudas son medidas que pueden mejorar la calidad de las observaciones en la escuela. Un profesorado capacitado tiende a realizar valoraciones más consistentes y fáciles de integrar con la información aportada por las familias y los profesionales sanitarios.

En conclusión, el estudio publicado brinda información valiosa sobre la percepción del déficit de atención desde dos contextos distintos. Incorporar en esta discusión el tema de la formación docente puede ayudar a entender mejor las diferencias entre informantes y aportar elementos para mejorar la identificación temprana del TDAH en el entorno escolar. En ese sentido, resulta un punto de interés también para los profesionales de atención primaria, ya que muchos padres acuden primero a este nivel y necesitan información clara y coherente sobre lo que se observa en la escuela. Con ello, se fortalecerá el proceso evaluativo y se facilitará un acompañamiento más adecuado para los estudiantes que presentan dificultades de aprendizaje.

## Financiación

La presente carta no ha recibido ningún tipo de financiación pública ni privada.

## Consideraciones éticas

Esta carta al editor no presenta resultados de investigación ni información proveniente de participantes humanos o animales. Por tal motivo, no fue necesaria la aprobación por parte de un comité de ética en investigación.

## Consentimiento informado

Dado que el contenido no incluye datos personales, historias clínicas ni testimonios de personas, no se requirió consentimiento informado.

## Declaración sobre el uso de inteligencia artificial

Durante la preparación de este manuscrito el autor utilizó la herramienta de inteligencia artificial ChatGPT (OpenAI) exclusivamente como apoyo para la redacción y la revisión del lenguaje. Posteriormente, el contenido fue revisado, editado y verificado de manera integral por el autor, quien asume la plena responsabilidad por la versión final del texto y su contenido científico.

## Contribución de los autores

El manuscrito fue concebido, redactado y revisado en su totalidad por el autor, quien aprueba la versión final y asume plena responsabilidad por su contenido.

## Conflicto de intereses

El autor declara que no existe ningún conflicto de intereses relacionado con el contenido de este manuscrito.
